# Crisis of Japanese Vascular Flora Shown By Quantifying Extinction Risks for 1618 Taxa

**DOI:** 10.1371/journal.pone.0098954

**Published:** 2014-06-12

**Authors:** Taku Kadoya, Akio Takenaka, Fumiko Ishihama, Taku Fujita, Makoto Ogawa, Teruo Katsuyama, Yasuro Kadono, Nobumitsu Kawakubo, Shunsuke Serizawa, Hideki Takahashi, Masayuki Takamiya, Shinji Fujii, Hiroyuki Matsuda, Kazuo Muneda, Masatsugu Yokota, Koji Yonekura, Tetsukazu Yahara

**Affiliations:** 1 Center for Environmental Biology and Ecosystem Studies, National Institute for Environmental Studies, Onogawa, Tsukuba, Ibaraki, Japan; 2 Nature Conservation Society of Japan, Shinkawa, Chuo-ku, Tokyo, Japan; 3 Tokushima Prefectural Museum, Bunka-no-mori Park, Hachiman-cho, Tokushima-shi, Tokushima, Japan; 4 Kanagawa Prefectural Museum of Natural History, Odawara, Kanagawa, Japan; 5 Faculty of Science, Kobe University, Kobe, Japan; 6 Faculty of Applied Biological Sciences, Gifu University, Yanagido, Gifu, Japan; 7 Department of Biology, Aichi Kyoiku University, Igaya-cho, Kariya, Aichi, Japan; 8 Hokkaido University Museum, Sapporo, Hokkaido, Japan; 9 Department of Environmental Science, Kumamoto University, Kumamoto, Japan; 10 Division of Human Environment, University of Human Environments, Okazaki, Aichi, Japan; 11 Faculty of Natural Environment and Information Sciences, Yokohama National University, Tokiwadai, Hodogaya-ku, Yokohama city, Kanagawa, Japan; 12 Faculty of Science, University of the Ryukyus, Senbaru, Nishihara, Nakagami, Okinawa, Japan; 13 Center for Academic Resources and Archives, Botanical Gardens, Tohoku University, Aoba, Aramaki, Aoba-ku, Sendai, Miyagi, Japan; 14 Department of Biology, Kyushu University, Higashi-ku, Fukuoka, Japan; University of Saskatchewan, Canada

## Abstract

Although many people have expressed alarm that we are witnessing a mass extinction, few projections have been quantified, owing to limited availability of time-series data on threatened organisms, especially plants. To quantify the risk of extinction, we need to monitor changes in population size over time for as many species as possible. Here, we present the world's first quantitative projection of plant species loss at a national level, with stochastic simulations based on the results of population censuses of 1618 threatened plant taxa in 3574 map cells of ca. 100 km^2^. More than 500 lay botanists helped monitor those taxa in 1994–1995 and in 2003–2004. We projected that between 370 and 561 vascular plant taxa will go extinct in Japan during the next century if past trends of population decline continue. This extinction rate is approximately two to three times the global rate. Using time-series data, we show that existing national protected areas (PAs) covering ca. 7% of Japan will not adequately prevent population declines: even core PAs can protect at best <60% of local populations from decline. Thus, the Aichi Biodiversity Target to expand PAs to 17% of land (and inland water) areas, as committed to by many national governments, is not enough: only 29.2% of currently threatened species will become non-threatened under the assumption that probability of protection success by PAs is 0.5, which our assessment shows is realistic. In countries where volunteers can be organized to monitor threatened taxa, censuses using our method should be able to quantify how fast we are losing species and to assess how effective current conservation measures such as PAs are in preventing species extinction.

## Introduction

Listing threatened species on the basis of IUCN Red List categories plays a prominent role in conservation practices. However, the practice has received criticism owing to its reliance on qualitative data [1] and insufficient data [2], [3], [4]. Quantitative analysis—for example, population viability analysis (PVA)—is seldom used in determining Red List categories, since time-series data are not available for most threatened species. As a result, conservation priorities have been determined only by the distribution of threatened species, without consideration of the probability of extinction of each species [5].

Protected areas (PAs) are a cornerstone of conservation efforts [6] and now cover nearly 11% (16 263 609 km^2^) of the world's land surface [7]. Various national governments have committed to expanding this coverage to 17%, as endorsed at the COP 10 of the Convention on Biological Diversity held at Nagoya in 2010 as one of the Aichi Biodiversity Targets [8]. The effectiveness of PAs should be assessed not only by their extent but also by to what degree they can reduce the risk of extinction of each species. However, assessments based on the risk of extinction of each taxon at a large scale, say the national scale, have not yet been conducted so far. The paucity of assessment of extinction risks based on data with high-enough spatial resolution hinders the effectiveness of assessment.

**Figure 1:**
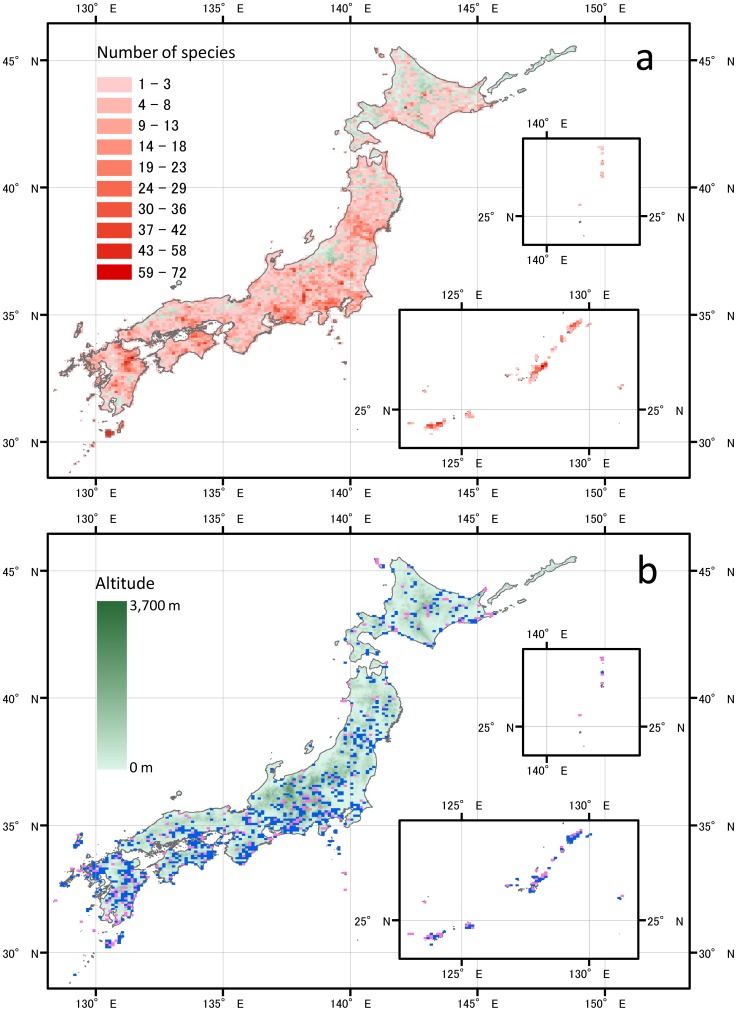
Distribution of 1618 vascular plant species in 4473 terrestrial cells (ca. 100 km^2^) in Japan, *a* and conservation prioritized cells, *b*. (a) One or more species were recorded in 3574 cells (79.9% of Japan). Green points represent the altitude of cells where no species were recorded with the same scale as in *b*. (b) Blue points represent 760 prioritized cells corresponding to 17% of the land area under the assumption that conservation effectiveness is 0.5 (see Fig. 2 and methods for details). Pink points represent 244 cells in which conservation effectiveness needs to be increased to conserve all threatened species; 237 of these are already included in the 760 prioritized cells. The maps were drawn using the ArcGIS 10.1 software (ESRI, Redlands, CA, USA).

To overcome these difficulties, we organized more than 500 lay botanists to monitor population sizes of 1618 plant taxa throughout Japan in ca. 100-km^2^ map cells (Fig. 1a). The population size and rate of change of at least one of those taxa were reported from 3574 cells (79.9% of Japan). Population size was determined on an ordinal scale of 1, 10, 100, 1000, or 10 000 individuals in 1994–1995 and 2003–2004, and changes between the census periods were used to quantify population decline. In addition, in both censuses, the rate of population decline during the prior decade was scored by the 500 lay botanists where possible. As the rate of population decline could differ among taxa and cells owing to differences in species and environmental characteristics, we need to consider not only the mean rate of population change but also its variance in order to evaluate the fate of threatened species. By taking advantage of the spatially intensive data, we quantified the uncertainty in the prediction of the viability of each taxon: we did not simply assume a constant trend for each population, but assumed that each population could be exposed to the rate of decline observed in other populations. Then, considering the uncertainty, we quantified the extinction risk for each taxon as a form of extinction probability using Monte Carlo simulations.

Using the time-series data, we also examined how effectively existing PAs can protect populations of threatened species from decline. In Japan, natural parks cover 14.3% (54 313 km^2^) of the nation [7]. However, only about half of this area (7.3%, 27 747 km^2^) has any regulation for biodiversity conservation and is managed by the national government. And only 0.91% (3449 km^2^) is strictly conserved as core zones within natural parks. Taking the actual conservation effectiveness of the existing PAs into account, we examined how much the global PA target of 17% could reduce the number of threatened species, defined here as those having a probability of extinction within the next 100 years of ≥10%. To do so, we created software that can find a (sub)optimized solution for a spatial arrangement of PAs that would reduce the overall extinction risk (reduction of extinction risk: RER) of all species.

## Methods

### Database of Japanese threatened vascular plants

More than 500 lay botanists participated in censuses in 1994–1995 and 2003–2004. The surveys were conducted across Japan in each ca. 100-km^2^ map cell (1° longitude by 40′ latitude). The population size (the number of reproductive individuals) at each cell was recorded as not present, 1–9, 10–99, 100–999 or ≥1000 in the first census; and as extinct, 1–9, 10–49, 50–99, 100–999, 1000–9999 or ≥10 000 in the second census. Furthermore, we asked participants to score the trend of decline (i.e., the proportion of the number in the previous period still alive 10 years later) if empirical evidence was available, as <1/100, 1/100–1/10, 1/10–1/2, 1/2–1, and >1 (i.e., increase).

Consequently, 1735 taxa were recorded in 3642 cells (81.4% of the total) that included at least one of the taxa. Of the 1735 taxa, 1618 that were recorded in 3574 map cells (79.9% of Japan) were quantifiable: Quantitative population sizes in both censuses were reported for 9115 populations covering 1089 taxa; quantitative population sizes in either census and information on decline were reported for 4755 populations covering 877 taxa; and quantitative population sizes in either census without a decline score were reported for 9600 populations covering 1184 taxa. In addition, decline scores were reported for 1413 populations of 437 taxa without population size data. In total, 24 833 populations covering the 1618 taxa were further analysed, as explained below. As some taxa consist of several populations which differ in the information types, they overlap among the information type categories. Most of the taxa had narrow ranges: 296 (18.3%) were found at only 1 cell, 374 (23.1%) at 2 or 3 cells, and 1086 (67.1%) at <10 cells.

### Probabilities of extinction of each taxon

The probabilities of the extinction of each of the 1618 taxa in each decade in the next century were computed by Monte Carlo simulations with 1000 replications.

The future population size of each taxon in cell *i* at time *t*+1, denoted as *N_i_*_(*t*+1)_, was estimated as
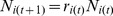
where *N_i_*_(*t*)_ is the number of individuals and *r_i_*_(*t*)_ is the rate of change in cell *i* at time step *t* (a multiple of 10 years).

Since the data set consists of size classes for population number and scores for rate of decline, and many records are incomplete, we considered uncertainty due to the data characteristics by including a stochastic process in choosing the initial population size, *N_i_*_(0)_, and the rate of change, *r_i_*_(*t*)_, for predictions.

For *N_i_*_(0)_, we randomly drew a value for each of 1000 replications from the recorded range of population size classes in the cell in each census (with values of 1000 for the class of ≥1000 and of 10 000 for the class of ≥10 000). Therefore, *N_i_*_(0)_ can be varied within the range of a size class recorded at cell *i* among replications.

To determine *r_i_*_(*t*)_ for each taxon, we assumed two pools from which *r_i_*_(*t*)_ could be drawn randomly for each time step (i.e., 10 years) for each replication: pool 1, which consists of records of population change in all cells where the taxon was reported; and pool 2, which consists of records of population change of all taxa in cell *i*. For each time step for each replication, a random value was drawn from pool 1 or pool 2 at a certain proportion (say 0.5 vs 0.5 from pool 1 and pool 2, respectively, such that the total probability for the two pools = 1.0) according to the scenario specified below.

Our records of the rate of change of population size are based on either (1) combinations of recorded population size classes in the first and second censuses in a cell, or (2) scores of the trend of decline (a change to <1/100, 1/100–1/10, 1/10–1/2, 1/2–1, and >1 of the value in the previous decade) reported by lay botanists. Where a type 1 record was available, we put it into the pools, treating the combination of size classes in both censuses as a single entity. Where a type 1 record was not available, we put a type 2 record into the pool as an entity instead.

The procedure to predict the probability of extinction for each taxon, based on the above settings, is as follows:

1. Determine initial population size in cell *i* by randomly drawing a value from the range of a size class (say 100–999) recorded in the 2003–2004 census in cell *i*.
2. Determine which pool is used to draw the rate of change according to a probability set *a priori* by a scenario specified following step 8, below.
3. Randomly draw an entity in the selected pool. If the drawn entity is a combination of population size classes (say 100–999 in the 1994–1995 census and 1–9 in the 2003–2004 census), then randomly draw a value for each census and calculate the rate of change at time *t* in cell *i* (*r_i_*_(*t*)_) by dividing the value in the second census by that in the first census. If the drawn entity is a score of the trend of population change (say 1/100–1/10), then determine *r_i_*_(*t*)_ by randomly drawing a value from the range in the entity.
4. Obtain *N_i_*_(*t*+1)_ by multiplying the population size, *N_i_*_(*t*)_, by *r_i_*_(*t*)_.
5. Perform steps 2 to 4 ten times (i.e., projection of population size over the next 100 years).
6. Perform steps 1 to 5 for all cells where the taxon was recorded.
7. Specify the time when the focal taxon goes extinct in all cells (i.e., the population size in every cell becomes <1).
8. Perform steps 1 to 7 one thousand times as replications and obtain a cumulative probability of extinction at time *t* as the proportion of the number of extinction events by time *t* in the 1000 replications.

**Figure 2:**
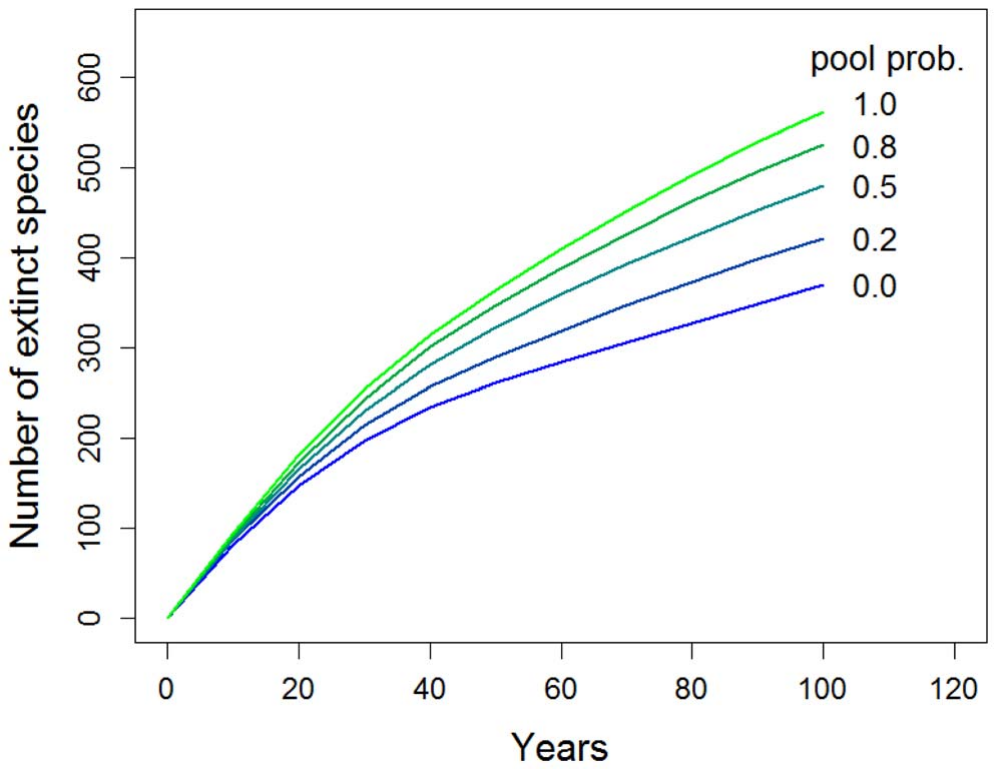
Number of extinct species predicted by the population viability analyses (PVAs) of 1618 vascular plant taxa in Japan. Results correspond to different scenarios in the choice of a rate of change class for the PVAs: classes were drawn (i) only from a pool of observed classes for each taxon in all of Japan (pool *P* = 1.0); (ii) from both the pool and the classes observed in the same cell at a certain ratio (*P* = 0.2–0.8); and (iii) only from those observed in the same cell (*P* = 0.0). Results from assumption (i) always gave the greatest rate of extinction.

For the selection of the pool at step 3, we assumed three different scenarios: (1) *r_i_*_(*t*)_ follows the whole-of-Japan pool of the observed rate of change for each taxon (i.e., always drawn from pool 1). (2) *r_i_*_(*t*)_ follows the rate of change observed for all of the taxa in the focal cell (i.e., always drawn from pool 2). (3) *r_i_*_(*t*)_ follows either the whole-of-Japan pool for each taxon or the locally observed rate of change (i.e., drawn from either pool 1 or pool 2) for all taxa at a probability of (pool 1 vs. pool 2) 0.2 vs 0.8, 0.5 vs 0.5, and 0.8 vs 0.2. The rationale of assumption 1 is that any change in a local population can happen to any other local population of that species. We call this the “what happened here may happen there” hypothesis. On the other hands, we call the assumption 2 as “what happened here will happen here again” hypothesis. Assumption 1 gives the greatest rate of extinction (Fig. 2). Although it may overestimate the risk, it makes the simplest and most precautionary assumptions about environmental and demographic stochasticity. We used this scenario in the subsequent prioritization analyses.

When we drew a value from a population size class or a score of the rate of population change, we used uniform probabilities on a logarithmic scale so as to make the expected *N_i_*_(*t*)_ or *r_i_*_(*t*)_ equal to the geometric mean of the upper and lower limits of the size and rate classes.

In the simulation, we assumed a few exceptions. When a population was not present in 2003–2004 in the field, the rate was defined as 1/10 000 so that any local populations go extinct during 10 years irrespective of their size. We ignored cells where population size was unknown in the second census. However, in cells where the population size was recorded in the first census, we started the simulation from the time of the 1994–1995 census. In this case, we performed step 5 eleven times to end the simulation at the same time as other populations. For 197 taxa which had populations with size information in either census but not enough information to calculate the rate of change in any population, we used the distribution of change rate classes obtained from all taxa.

### Assessment of effectiveness of existing protected areas

**Figure 3:**
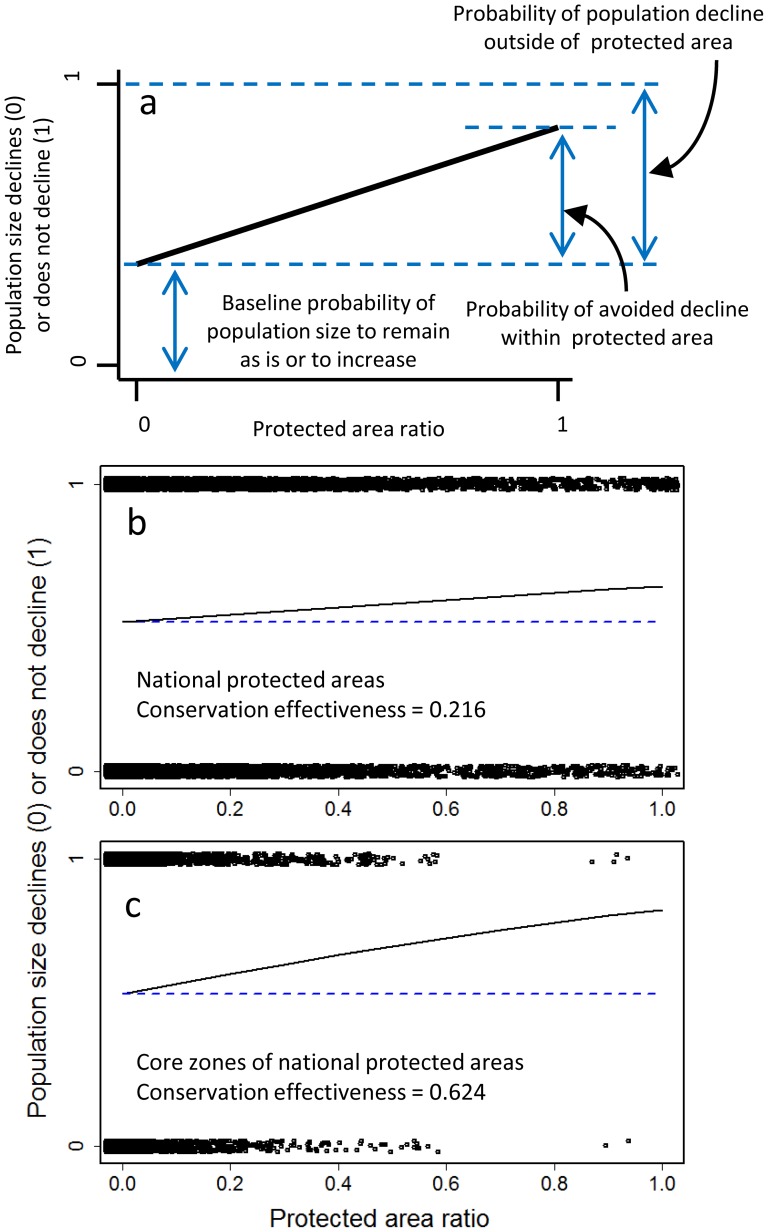
Relationships between the ratio of protected area in a cell and local population decline (defined as either decreased [0] or constant or increased [1]). (a) Conservation effectiveness is calculated as the ratio of the probability of avoided decline within a protected area to the probability of population decline outside the protected area. (b) Empirical assessment using total protected area, which corresponds to natural parks with regulations for biodiversity conservation (27 747 km^2^: ca. 7.3% of Japan). (c) Empirical assessment using core zones of the national parks (3449 km^2^: ca. 0.91% of Japan). Dark blue dashed line represents baseline probability of avoided decline in cells where protected area = 0%.

Natural parks cover 14.3% (54 313 km^2^) of Japan [7]. However, only about half of this area (7.3%, 27 747 km^2^) has any regulation for biodiversity conservation and is managed by the national government. And only 0.91% (3449 km^2^) is strictly conserved as core zones within natural parks, in which no human disturbance is permitted. In combination with the time-series data, we assessed how effectively existing PAs can protect populations of threatened species from decline. Because the exact locations of populations within a map cell were not recorded in the censuses, we assessed the effectiveness of the PAs as follows. First, we divided all records of the rate of change of local populations between “decreased” and “constant or increased”. Then we related the category to the proportion of protected area in a cell by logistic regression. From these results, we divided the probability of avoided decline within a protected area by that of population decline outside of protected area (Fig. 3a). The result is the probability of protection success, or “conservation effectiveness”. We also compared performance in terms of Akaike's information criterion (AIC) between the logistic regression which has proportion of protected area as an explanatory variable and the null model i.e., a logistic regression which has only a constant term. As an assessment of current state of populations within PAs at a whole nation scale, we were just interested in how populations within PAs have lower probability of decline than those outside of PAs. Therefore we did not include any factors other than coverage of PAs in the regression analysis.

### Spatial optimization of the reserve network to reduce overall extinction risks

#### Development of Spatial Prioritizer based on Extinction Risks (SPERS)

We developed a tool for the prioritization of spatial conservation, finding a (sub)optimized solution of PAs to reduce the overall risk of extinction (reduction of extinction risk: RER) of all species. RER is defined as:
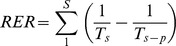
where *S* is the total number of target taxa and *T_s_* and *T_s-_*_p_ are the expected waiting times for extinction without and with protection, respectively, for a taxa *s*., which are quantified through the PVA simulations explained above. Our tool finds a spatial arrangement that maximizes the RER under a given number of PAs using a so-called “greedy” algorithm [9] to consider the conservation effectiveness of reserves. The maximization of the RER by a simple and honest algorithm will easily reach an infeasible computation time. This is because we must re-evaluate the risk of extinction of every species using the PVA simulation at every time when a new reserve is added. We make this feasible by using an efficient computational procedure, named SPERS (Spatial Prioritizer based on Extinction Risks).

Schematic algorithms of SPERS are shown in Figures S1–S3. The first component of SPERS prepares a database of the probabilities of local extinction (Fig. S1). SPERS calculates the probability of extinction of a local population of each taxon in each cell using the same procedure as for PVA, except that it considers conservation effectiveness. To do so, it draws on two databases of probabilities of local extinction: with no protection and with protection at an arbitrary probability of success (i.e., conservation effectiveness set *a priori*) (Fig. S2). We assumed conservation effectiveness values of 0.2, 0.5, 0.8 and 1.0 on the basis of the results of our empirical assessments explained in the previous section. When calculating the probabilities of local extinction with protection, if the rate of change of a taxon in a protected cell is *r_i_*(*t*) <1, SPERS alters it to 1 (no population change) with a probability that corresponds to conservation effectiveness set *a priori*.

The second component of SPERS calculates the risk of total extinction with a given set of protected sites (Fig. S2). It uses the two databases of the probabilities of local extinction in the first component. Selecting one database according to the input set of protected sites, SPERS can evaluate the local extinction of species at each site, which collectively represents the risk of total extinction of a species with the given protected sites. By omitting the re-evaluation of the risk of local extinction through the use of the two database, SPERS can efficiently evaluate the risk of total extinction in a set of protected sites.

The final component of SPERS finds an arbitrary number of sites to be protected to minimize total extinction risk (i.e., maximize RER) (Fig. S3). Relying on a greedy algorithm, SPERS incrementally adds a new site that is the most effective in terms of RER to reach the given number of sites. Although the solution obtained by the greedy algorithm is not guaranteed to be optimal, the algorithm is known to provide adequate approximation of optimal solutions [9].

#### Performance comparisons with existing methods

We compared the performance among spatial prioritization methods in terms of RER. As comparators, we used complementarity analysis and hotspot analysis [10]. In complementarity analysis, cells are incrementally selected using a greedy algorithm to maximize the number of represented species in the protected areas; after all species are represented in at least one cell, the algorithm proceeds so that all species are represented in more cells. In hotspot analysis, or scoring analysis, cells are incrementally selected according to species richness in them. In addition, we compared the performance of those methods with random selection, in which cells were added randomly to protected areas.

#### Spatial prioritization analysis using SPERS

We applied SPERS to our data, which included 757 threatened taxa, defined here as those having a probability of extinction within the next 100 years of ≥10% in the PVA simulations. First, we quantified how the RER increased as protected areas increased under an assumption of perfect conservation effectiveness (i.e., conservation effectiveness = 1.0). In this case, when we prioritized a cell, if *r_i_*_(*t*)_<1 at a conserved cell, it was always altered to 1 (no population change).

Then, according to the result of the assessment of conservation effectiveness, we examined how the RER increased as protected areas increased under the more realistic assumption of imperfect conservation effectiveness of 0.8, 0.5 and 0.2. In these cases, when we prioritized a cell, if *r_i_*_(*t*)_<1 at a conserved cell, it was altered to 1 (no population change) with a probability of 0.8, 0.5, and 0.2, according to the assumed conservation effectiveness. In each case, we examined to what degree 17% of protected areas (i.e., the Aichi target) could reduce the risk of extinction of the 757 threatened taxa.

## Results & Discussion

Our PVA projected that between 370 and 561 of the 1618 taxa will go extinct during the next century (Fig. 2). The worst scenario corresponded to the assumption that “what happened here may happen there” rather than “what happened here will happen here again”. In contrast, only 42 taxa were lost during the past 60 years (Table 1). In Japan, 7087 vascular plant taxa have been recorded [10], making the recent extinction rate only 0.01% per year. The projected extinction rate, in contrast, is 0.05% to 0.08% per year. The number of seed plants lost in the world from 1990 to 1992 was estimated to be 163 species [11], or 0.03% per year for the 240 000 described species [12]. The projected extinction rate in Japan is two to three times this rate.

**Table 1:** List of vascular plant species categorized as Extinct (EX) or Extinct in the Wild (EW) in the Red List compiled by Ministry of the Environment of Japan in 2012.

Scientific name
**Extinct (EX)**
*Lycopodium cunninghamioides*
*Botrychium boreale*
*Ophioglossum parvifolium*
*Hypolepis tenuifolia*
*Asplenium austrochinense*
*Arachniodes yasu-inouei* var. *angustipinnula*
*Tectaria dissecta*
*Pyrrosia angustissima*
*Elatostema lineolatum* var. *majus*
*Ranunculus gmelinii*
*Rubus hatsushimae*
*Flemingia strobilifera*
*Lespedeza hisauchii*
*Euphrasia insignis* subsp. *insignis* var. *omiensis*
*Euphrasia insignis* subsp. *insignis* var. *pubigera*
*Euphrasia multifolia* var. *kirisimana*
*Cirsium toyoshimae*
*Aletris makiyataroi*
*Burmannia coelestris*
*Thismia tuberculata*
*Eriocaulon cauliferum*
*Eriocaulon seticuspe*
*Carex disperma*
*Cyperus diaphanus*
*Cyperus procerus*
*Fimbristylis leptoclada* var. *takamineana*
*Fimbristylis pauciflora*
*Acanthophippium striatum*
*Neottia kiusiana*
*Odontochilus poilanei*
*Renanthera labrosa*
*Zeuxine boninensis*
**Extinct in the wild (EX)**
*Thelypteris aurita*
*Dryopteris shibipedis*
*Magnolia pseudokobus*
*Kalanchoë spathulata*
*Malus hupehensis*
*Astragalus sikokianus*
*Viola stoloniflora*
*Lycoris sanguinea* var. *koreana*
*Eriocaulon heleocharioides*
*Liparis uchiyamae*

An ideal assessment is based on spatially extensive, temporally intensive data. Although our assessment is spatially extensive, it is limited to two time points at which abundances were estimated on an ordinal scale. As this may cause uncertainty in the estimation of the risk of extinction of each taxon, we considered the uncertainty due to the limited time-series data by incorporating stochasticity in the predictions of extinction probability for each taxon: instead of directly using the observed rate of population change to predict population dynamics for a taxon at a location, we pooled the observation records across either taxa or locations and assumed that the rate of population change, *r*, can vary even within a cell among time steps and replications according to the frequency distribution of the records in the pools. This would be the most honest way to reflect the data uncertainty in the results of predictions and the most efficient way to take advantage of the spatially high-resolution data. As we aggregated the results of all 1618 taxa in estimating the rate of extinction of all taxa, the results are likely to capture general trends in spite of the uncertainty at each taxon level.

However, because these pooled data are themselves constrained by the temporal trend of 1994–1995 and 2003–2004, if this trend had been unique in comparison to those of the other time intervals, future projection would be biased. Therefore the predictions should be updated and improved through subsequent assessments, and we are now planning the third nationwide census for the near future. There is now an urgent need to quantify the risk of extinction of species in rapid decline by a scientifically sound method so as to facilitate awareness of the crisis and to prompt decision makers to act to prevent extinction. That is, it is important to act now, even based on imperfect data, to prevent the loss of species that would become extinct while we wait for better data to become available. This study will help meet these needs.

In the assessment of the probability of protection success, or “conservation effectiveness”, compared with the null model, which assumes no effect of the PAs, models that included PAs (Fig. 3b) and core zones of PAs (Fig. 3c) were superior (i.e., AIC was smaller by 40 and 20, respectively), which suggests that existing PAs improve the status of populations to some extent. However, even in the core zones, conservation effectiveness was only 0.624 (Fig. 3c), indicating that the effectiveness of the PAs was far from perfect.

**Figure 4:**
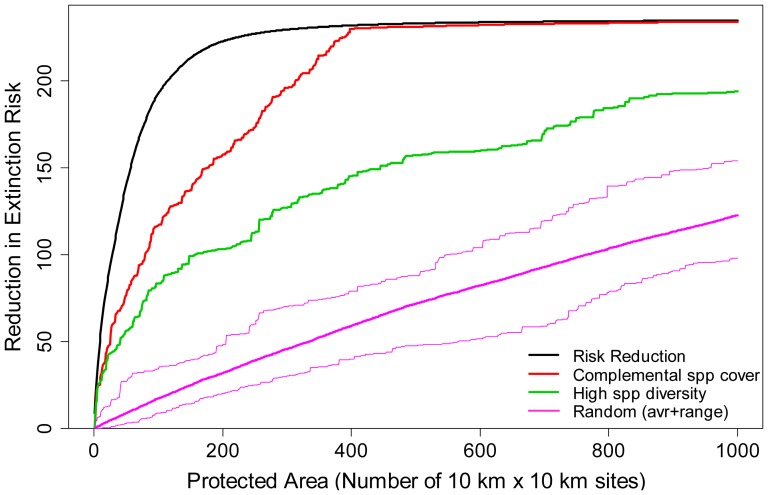
Comparison of performance among spatial prioritization methods in terms of reduction in extinction risk. “Risk Reduction”: cell selection using SPERS. “Complemental spp. cover”: complementarity analysis in which cells are incrementally selected using a greedy algorithm to maximize the number of represented species in the protected areas; after all species are represented in at least one cell, the algorithm proceeds so that all species are represented in more cells. “High spp. diversity”: hotspot analysis in which cells are incrementally selected according to species richness in them. “Random”: cells are added randomly to protected areas and the random selection is performed 100 times; thick pink curve  =  mean, thin pink curves  =  maximum–minimum range.

**Figure 5:**
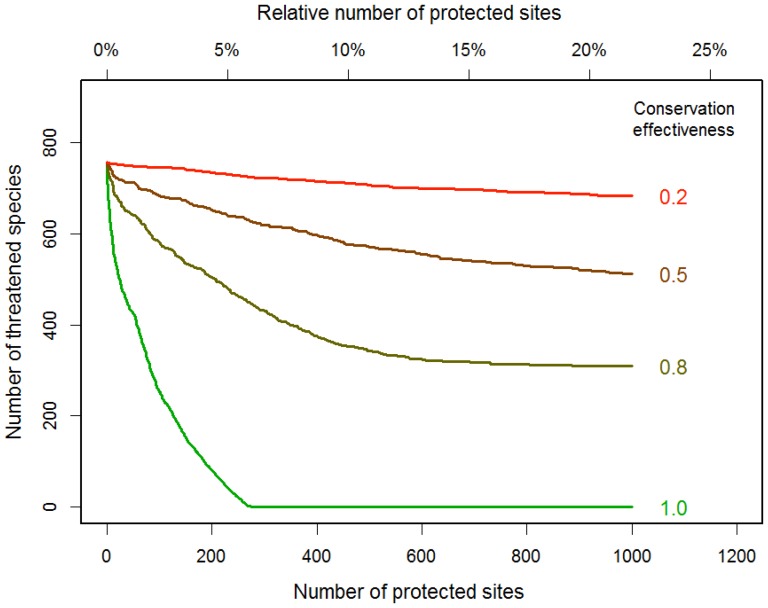
Relationships between number of protected cells and number of species remaining as threatened (probability of extinction in the next 100 years ≥10%). Cells are selected to maximize the reduction of extinction risk (see text for details).

Our spatial conservation prioritization tool, SPERS, proved more efficient than the conventional complementarity analysis and hotspot analysis, especially when the number of protected cells was less than ideal so that not all taxa were represented in PAs (Fig. 4). Considering that the resources available for conservation are always limited and the extent of the protected area is often far from enough, our method is likely to outperform others in most situations. SPERS showed that the optimized PAs could reduce extinction risks effectively, in that protecting 250 cells (ca. 5.6% of Japan) rendered all of the endangered species non-threatened (Fig 5). However, when we considered more realistic conservation effectiveness values, the total RER was reduced rapidly: under the assumption of 17% PAs (i.e., protecting 760 cells), 443 (58.5%) of the 757 threatened taxa become non-threatened at an effectiveness value of 0.8, 221 (29.2%) at a value of 0.5, and only 64 (8.45%) at a value of 0.2 (Fig. 5). In these cases, even if we could achieve the 17% target with an optimized PA selection (including the relocation of existing PAs), we may be unable to achieve the target. These proportions are also below the level set by the global strategy for plant conservation endorsed at COP 10, which requires “at least 75 per cent of known threatened plant species conserved in *situ*”. In addition, the relative importance of conservation effectiveness will increase as the number of protected cells is increased (beyond about 200 cells [4.5%] in this case; Fig. 5). As the spatial arrangement of existing protected sites is likely to deviate from the optimum [13], these results may still overestimate the overall effectiveness of PAs.

**Figure 6:**
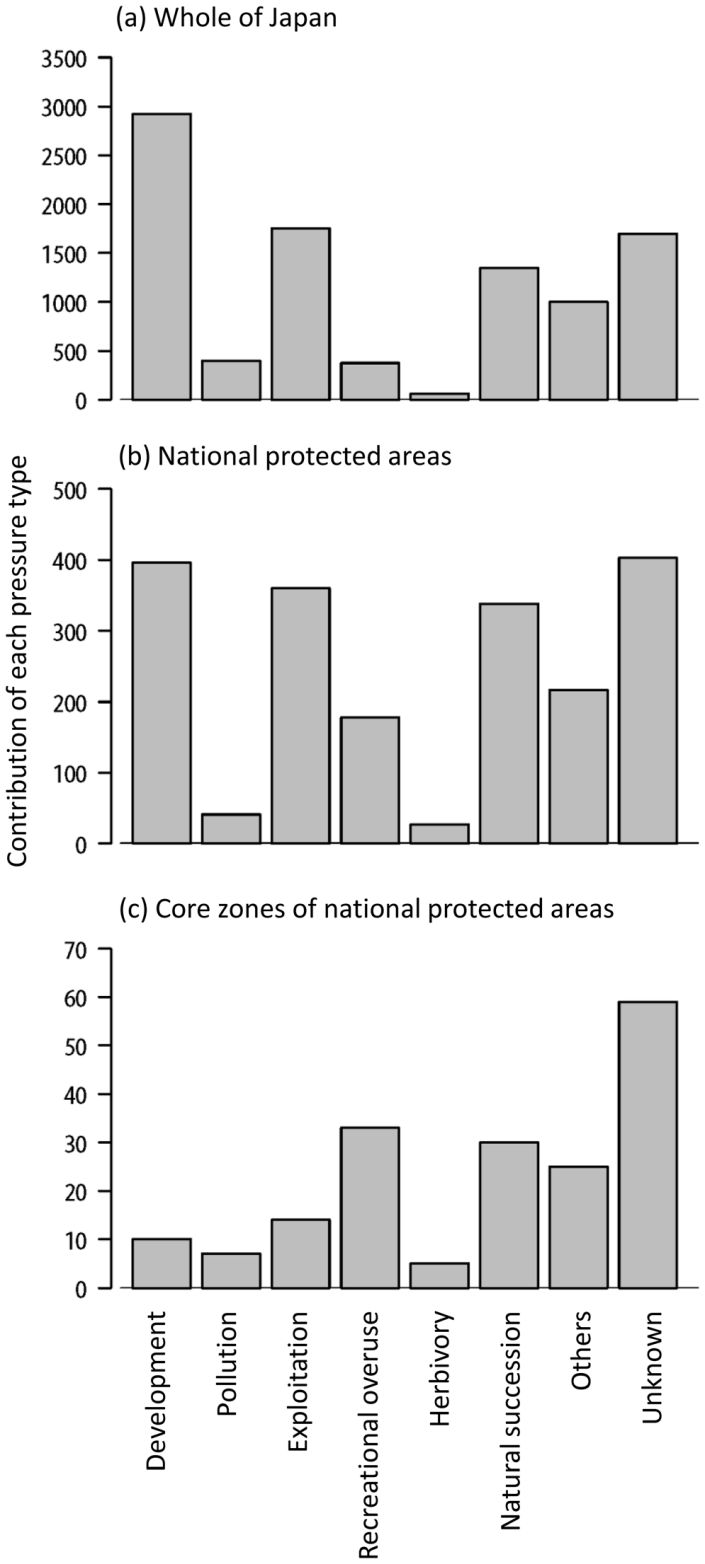
Contribution of pressure types causing decline of local populations in (a) whole of Japan, (b) national protected areas (cells with >20% of protected areas) and (c) core zones of national protected area (cells with >20% of core zones). For each combination of pressure type and species, the number of cells with declining population was calculated, and the numbers are summed for each pressure type.

Our results demonstrate that mass extinction of plants is not an exaggerated scenario but a realistic possibility even in a developed country such as Japan, where conservation efforts are intensive. Under the realistic conservation effectiveness for each PA, the 17% target is likely to be inadequate. When the average conservation effectiveness is around 0.5, which our assessment shows is realistic, then to make the network of PAs adequately effective, we must improve the conservation effectiveness in about a third of the cells (244 of 760) to 1.0 (i.e., perfect conservation; Fig. 1b). This necessity indicates the need to improve the conservation effectiveness of PAs, in addition to extending the overall coverage of PAs. To improve the effectiveness of conservation areas, we need to know the types of pressures causing population decline in the areas. The data set records types of pressures which are suspected to be causing a population decline for each species in each cell. For each combination of pressure type and species, we summed the number of cells with declining population, then we summed the number of cells for each pressure type. The larger the sum, the greater the pressure on the plants. We carried out these calculations for all of Japan, national protected areas, and core zones of the protected areas. Among the pressures, land development and exploitation were less important at cells with core zones of PAs, whereas pressures of recreational overuse, herbivory and vegetation succession were increased or not improved (Fig. 6). Conservation effectiveness could be further improved by developing management schemes to focus on specific pressures. For the 17% target to be adequate, the protection success must be greatly increased in each PA or in the PAs that show the greatest risk of extinction for a given species.

As far as we know, this is the world's first reliable quantification of plant species loss and quantitative assessment of the adequacy of the 17% Aichi Biodiversity Target based on time-series population data at a national scale. Monitoring data collected by the public can play an essential role in assessing biodiversity [14], [15]. Although the information we obtained covers changes only in population size classes, our approach provides a practical and quick solution to evaluating the fates of thousands of threatened species from a set of time-series observations. It should provide a basis for similar efforts to quantify species loss in other countries.

## Supporting Information

Figure S1 **Preparation of database of probabilities of local extinction in SPERS.**
(PDF)

Figure S2 **Estimation of extinction risks under the given set of protected sites in SPERS.**
(PDF)

Figure S3 **Selection of** ***N*** **sites to be protected to minimize the total extinction risk in SPERS.**
(PDF)
