# Correction: Crisis of Japanese Vascular Flora Shown By Quantifying Extinction Risks for 1618 Taxa

**DOI:** 10.1371/journal.pone.0102384

**Published:** 2014-07-03

**Authors:** The PLOS ONE Staff

There is an error in the “Acknowledgements” and “Financial Disclosure” statements. Please refer to the correct statements below:

Acknowledgments:

We thank the more than 500 lay botanists who contributed to the Japanese Red Data Book project directed by the Japanese Society for Plant Systematics. We also thank Kazuo Somiya, of the Ministry of the Environment of Japan, and Yasuhiro Ibaragi, Masato Nagatsu, and Kaori Sato, of the Japan Wildlife Research Center, for their help and encouragement during the project. This work was supported by the

Ministry of the Environment of Japan and the Japanese Society for Plant Systematics.

Financial Disclosure:

This work was supported in part by the Environment Research and Technology Development fund (S9-1) of the Ministry of the Environment, Japan. The funders had no role in study design, data collection and analysis, decision to publish, or preparation of the manuscript.
