# Supplementary figures and images for: Crisis of Japanese Vascular Flora Shown By Quantifying Extinction Risks for 1618 Taxa
Source: PLoS One. 2014 Jun 12;9(6):e98954. doi: 10.1371/journal.pone.0098954 (PMC4055661; doi:10.1371/journal.pone.0098954)

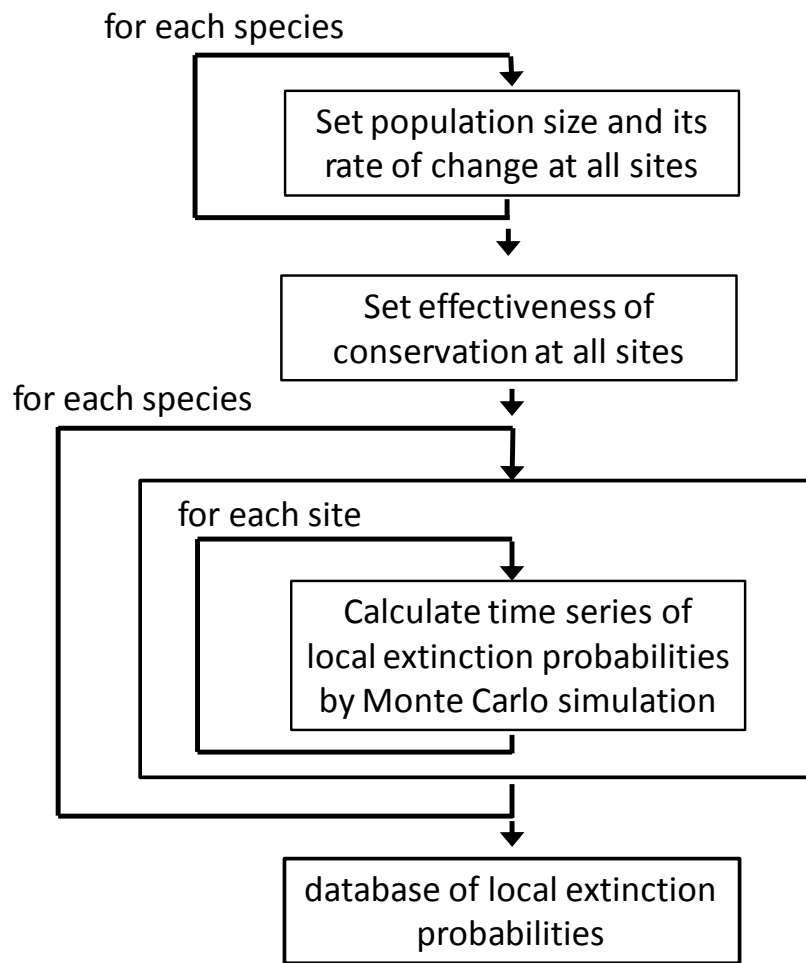

Figure S1 Preparation of database of probabilities of local extinction in SPERS.

Supplement: Figure S1 — Preparation of database of probabilities of local extinction in SPERS. (PDF) [file pone.0098954.s001.pdf]

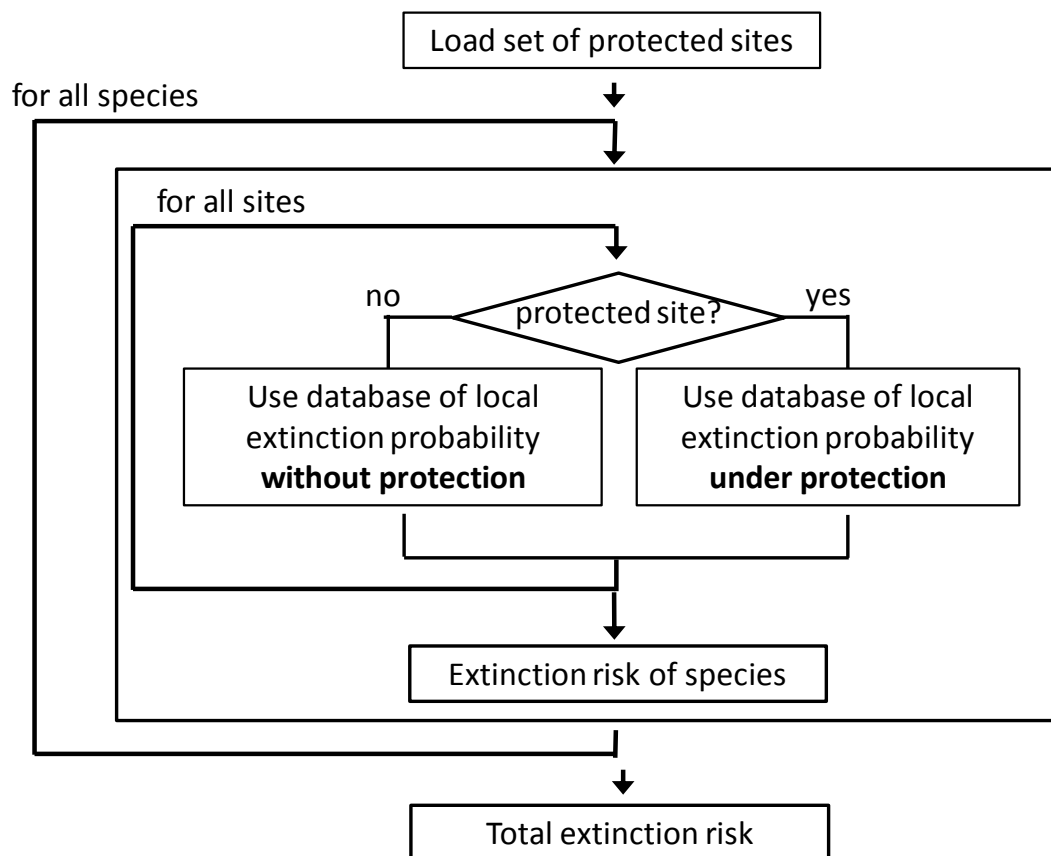

Figure S2 Estimation of extinction risks under the given set of protected sites in SPERS.

Supplement: Figure S2 — Estimation of extinction risks under the given set of protected sites in SPERS. (PDF) [file pone.0098954.s002.pdf]

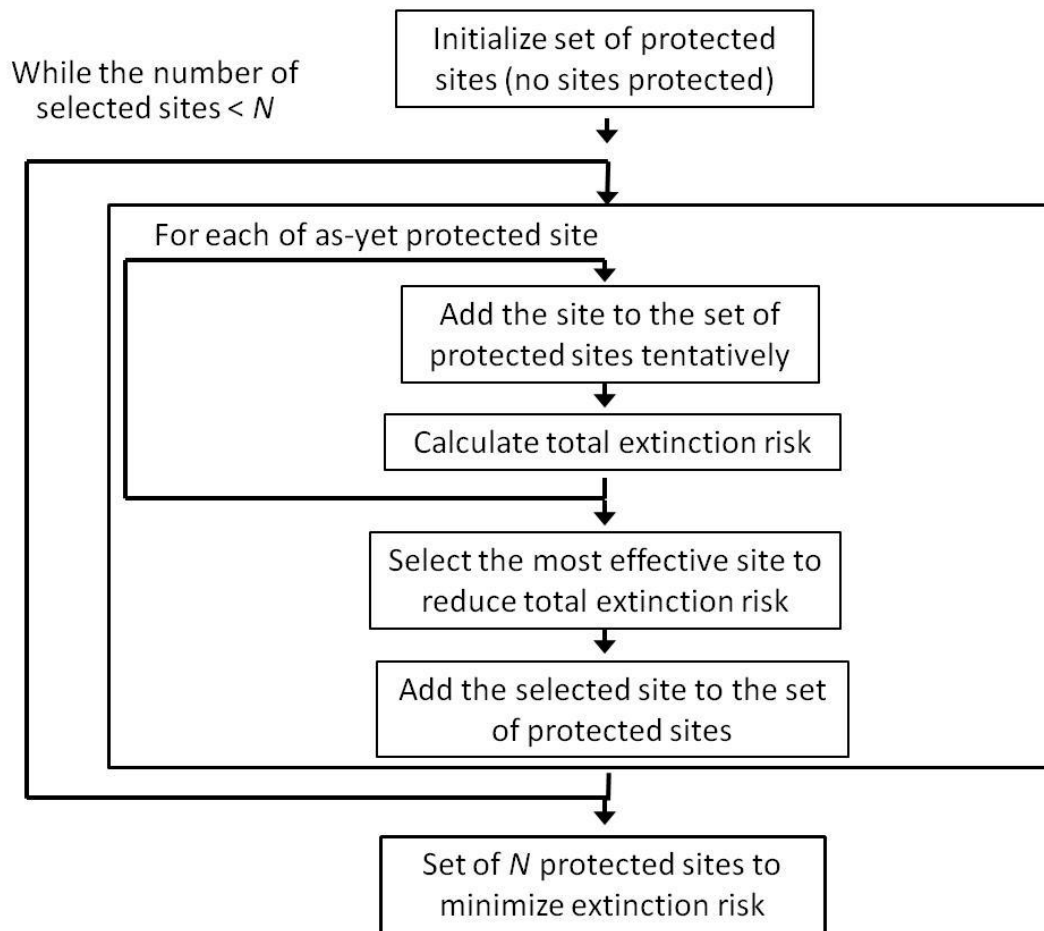

Figure S3 Selection of  $N$  sites to be protected to minimize the total extinction risk in SPERS.

Supplement: Figure S3 — Selection of N sites to be protected to minimize the total extinction risk in SPERS. (PDF) [file pone.0098954.s003.pdf]
